# Levels of plasma neurofilament light chain and cognitive function in patients with Alzheimer or Parkinson disease

**DOI:** 10.1038/s41598-018-35766-w

**Published:** 2018-11-26

**Authors:** Yung-Shuan Lin, Wei-Ju Lee, Shuu-Jiun Wang, Jong-Ling Fuh

**Affiliations:** 1 Department of Neurology, Neurological Institute, Taipei Veterans General Hospital, Taipei, Taiwan; 2 Faculty of Medicine, National Yang-Ming University School of Medicine, Taipei, Taiwan; 3 Neurological Institute, Taichung Veterans General Hospital, Taichung, Taiwan; 4 Dementia and Parkinson’s Disease Integrated Center, Taichung Veterans General Hospital, Taichung, Taiwan; 5 Center for Geriatrics and Gerontology, Taichung Veterans General Hospital, Taichung, Taiwan; 6 Brain Research Center, National Yang-Ming University, Taipei, Taiwan

## Abstract

Plasma neurofilament light (NFL) has been proposed as a blood-based biomarker for neurodegeneration in Alzheimer’s disease (AD) and parkinsonian disorders. However, the relationship between plasma NFL and cognitive decline in dementia due to Parkinson’s disease (PD) remains to be elucidated. In this research, 119 AD, 56 mild cognitive impairment (MCI), 26 non-demented PD (PDND), and 23 Parkinson’s disease dementia (PDD) patients, as well as 59 cognitively healthy controls (HC) were recruited. Each subject underwent a battery of neuropsychological testing. Plasma NFL levels were measured in duplicate using an NF-Light assay and transferred onto the Simoa platform with a home-brew kit. Plasma NFL was significantly increased in the AD group, compared with the control, MCI, PDND, and PDD groups. Plasma NFL was significantly higher in the PDD group, compared with the PDND group. High plasma NFL correlated with poor cognition in AD and PD, but not with motor symptoms in PD. Plasma NFL may represent a biomarker of cognitive decline in AD and PD, with more specificity for AD.

## Introduction

Neurofilament light chain (NFL) is one of the three subunits of neurofilaments, which are specific cytoskeletal proteins of neurons and particularly abundant in largely myelinated axons. Axonal damage releases NFLs into cerebrospinal fluid (CSF) and eventually into blood. Higher NFL levels are believed to represent more severe cerebral axonal degeneration. NFL levels have been linked to several neurodegenerative diseases, such as Alzheimer’s disease (AD)^1^, Parkinson’s disease (PD), atypical parkinsonian disorders (APD)^2^, frontotemporal dementia (FTD)^3^, amyotrophic lateral sclerosis (ALS)^4^, Huntington disease (HD)^5,6^, and multiple sclerosis (MS)^7^.

In mouse models, NFL levels in CSF and blood increased in association with the existence of α-synucleinopathy, tauopathy, and β-amyloidosis. In addition, blood and CSF NFL levels were strongly correlated, and NFL increases coincided with the onset and progression of proteopathic brain lesions^8^. Clinical studies also demonstrated that patients with mild cognitive impairment (MCI) or AD dementia with Aβ pathologic features have higher plasma NFL^1^. As for parkinsonian disorders, one recent study showed blood NFL concentration can be used to distinguish PD from APD^2^; significantly increased levels were observed in patients with multiple system atrophy (MSA), progressive supranuclear palsy (PSP), or cortical basal syndrome (CBS), compared to patients with PD, as well as healthy controls. However, to our knowledge, no previous study has investigated blood NFL concentrations in non-demented PD (PDND) *vs* Parkinson disease’s dementia (PDD).

Neuropsychological tests, neuroimaging, and CSF biomarkers may be used to diagnose cognitive decline in various syndromes that involve dementia. However, the use of these markers is hampered by lack of sensitivity at early stages of disease, high cost, and invasiveness. A readily available blood-based biomarker is needed for efficient monitoring of disease progress and could be used as a screening tool in primary care. When Simoa method, rather than ELISA or electrochemiluminescence immunoassay, is used to measure plasma NFL, levels correlate strongly with those of CSF NFL^9^. As a potential indicator of disease severity or prognosis, serial measurements of plasma NFL may be considerably more practical than measurements of CSF NFL, which are inherently invasive.

In this study, we sought to investigate whether plasma NFL levels could be used as a reliable biomarker of disease severity and prognosis in patients with MCI, AD, PDND, or PDD. We expected that plasma NFL concentration would be elevated in patients with dementia due to AD or PD, compared with healthy controls. We hypothesized that concentrations would vary between PDND and PDD, and that increased plasma NFL levels would reflect disease severity.

## Methods

### Participants

We recruited normal healthy controls (HC), as well as amnesic MCI, AD, and PD patients seen at the outpatient clinics of Taipei Veterans General Hospital. HC were volunteers with normal cognitive function. AD diagnoses required multidisciplinary consensus, according to the clinical criteria for probable AD as described by the National Institute on Aging-Alzheimer’s Association^10^. A diagnosis of MCI was made according to the revised consensus criteria from 2004^11^. The cut-off value for diagnosis of MCI was set at 1.5 standard deviations below the age-adjusted norm for Wechsler Memory Scale III logical memory test^12^. Inclusion criteria for PD adhered to those proposed by the UK Parkinson’s Disease Society Brain Bank^13^. PD patients were further divided into PDND and PDD. PDD was diagnosed when patients fulfilled criteria proposed by the Movement Disorder Society Task Force^14^. Patients who had developed dementia within 1 year after PD onset were excluded. We also excluded patients with cognitive and behavioral symptoms presenting as a result of other conditions, including acute confusion due to systemic disease/abnormality or drug intoxication, major depressive disorder according to the Diagnostic and Statistical Manual of Mental Disorders 5^th^ edition (DSM-V), probable vascular dementia, normal pressure hydrocephalus, progressive supranuclear palsy, or history of significant head trauma followed by persistent neurologic deficit or known structural brain abnormality. Disease duration was defined as the period between the initial onset of symptoms (as reported by the caregiver) and participation in the study. Informed consent was obtained from all patients and their caregivers before participation in the study. This research project was approved by the institutional review boards at Taipei Veterans General Hospital.

### Clinical evaluation and procedures

All patients received a standardized evaluation that included physical examination, clinical interview, neuropsychological assessment, laboratory tests, and either brain magnetic resonance or computed tomographic imaging. Height and body weight were measured to calculate body mass index (BMI). Cognitive function was assessed with standard procedures. The Min-Mental State Examination (MMSE)^15^ was used to assess global cognition. The Clinical Dementia Rating (CDR)^16^ was administered to determine the severity of dementia. The 12-item memory test^17^, modified 15-item Boston Naming Test^18^, category verbal fluency test^19^, and forward and backward digit span test^20^ were used to assess short-term memory, language, executive function, attention, and working memory, respectively. The UPDRS^21^ was used as a measurement of clinical PD severity.

### Measurement of plasma NFL levels

Freshly drawn venous blood was collected at baseline in tubes containing ethylenediaminetetraacetic acid (EDTA). Samples were then centrifuged and stored in polypropylene tubes at −80 °C until biochemical analysis. Plasma NFL levels were measured in duplicate using Quanterix SIMOA kits, according to the manufacturer’s instructions and standard procedures. All sample coefficients of variance (CVs) of duplicate measurements were below 18.5%.

### DNA analysis

Genomic DNA was isolated from whole blood using a Gentra Puregene kit, according to the manufacturer’s protocol (Qiagen, Hilden, Germany). Presence of the ε2, ε3, and ε4 alleles of the Apolipoprotein E *(APOE*) gene were determined by genotyping of SNPs rs429358 and rs7412. An *APOE* ε4 carrier was defined as having at least one ε4 allele (including ε2/ε4, ε3ε4, and ε4/ε4). Genotyping of rs429358 and rs7412 was performed using the TaqMan genotyping assay (Applied Biosystems, Foster City, CA, USA). Polymerase chain reaction was performed in 96-well microplates with an ABI 7500 real-time PCR machine (Applied Biosystems). Allele discrimination was achieved by detecting fluorescence using System SDS software version 1.2.3 (Applied Biosystems).

### Statistical analysis

Because the skewness and kurtosis values of plasma NFL measurements exceeded 1, we log-transformed these values prior to analysis. The Chi-square test, independent two-sample t-test, and one-way analysis of variance with post-hoc Tukey test were used to identify differences between control and patient groups in terms of demographic, clinical, and neuropsychiatric variables. One-way analysis of covariance was used to examine differences in plasma NFL levels between controls and patient groups after adjusting for age and sex, year of education and *APOE* ɛ4 carrier status. Pearson correlation analysis was used to assess the relationship between plasma NFL levels and age, as well as correlations between plasma NFL levels, MMSE score, and UPDRS. Independent *t*-test was used to examine the difference of NFL levels between *APOE* ɛ4 carriers and non-carriers as well as between stable MCI patient and MCI conversion to AD patient during follow-up. We used Cox regression model adjusting for age, sex, years of education, disease duration, and *APOE* ɛ4 carrier status to evaluate the association of NFL with MCI conversion to AD. All statistical analyses were performed with SPSS software, version 18.0 (IBM, Inc., Armonk, NY, USA), with p < 0.05 used to indicate statistical significance.

### Ethics approval and consent to participate

Informed consent was obtained from all patients and their spouse or offspring who are legal representatives of the patient prior to participation in the study. This research project was approved by the institutional review boards at Taipei Veterans General Hospital (IRB number 2012-05-033B).

### Consent for publication

Written informed consent was obtained from the patient for publication of this research article. All authors have approved the manuscript for submission and gave consent for publication.

## Results

### Subjects and demographics

Demographic features of study participants are presented in Table 1. There were no significant differences across groups (HC, MCI, AD, PDND, and PDD) in terms of sex or BMI. Age differed significantly across groups, with post-hoc comparisons revealing younger age in participants with PDND compared to controls, as well as patients with MCI, AD, or PPD (p < 0.001, p < 0.001, p < 0.001, p = 0.004, respectively). Post-hoc analysis showed that patients with PDD or AD had years of education compared to controls in the post hoc analysis (p = 0.002 and 0.001 respectively). UPDRS motor scores differed significantly between PDND and PDD groups, with worse motor severity in participants with PDD compared to those with PDND (p = 0.005).

**Table 1:** Demographic data of study participants.

	AD (n = 119)	MCI (n = 56)	PDND (n = 26)	PDD (n = 23)	HC (n = 59)	P-value
Male patients*	56 (47.1%)	27 (48.2%)	13 (50%)	14 (60.9%)	31 (52.5%)	0.79
Age, year	77.3 (5.1)	76.0 (5.6)	69.6 (10.8)	76.3 (9.1)	77.0 (6.2)	<0.001^a^
Years of education	9.5 (4.8)	11.0 (3.7)	10.7 (5.5)	8.0 (5.1)	12.4 (5.0)	<0.001^b^
Body mass index	24.0 (3.2)	23.6 (2.8)	25.2 (3.5)	24.6 (3.1)	24.4 (3.0)	0.22
*APOE* ε4 carrier*	42 (35.3%)	13 (23.2%)	4 (17.4%)	3 (14.3%)	7 (12.1%)	0.007
**Clinical dementia rating scale**						
0.5	14	56	—	—	—	—
1.0	74	—	—	—	—	—
2.0	26	—	—	—	—	—
3.0	5	—	—	—	—	—
UPDRS	—	—	17.6 (10.1)	30.9 (19.5)	—	0.005
MMSE	18.6 (6.2)	26.4 (2.3)	26.9 (2.6)	23.3 (4.6)	27.8 (2.1)	<0.001^c^
Delayed-recall	1.6 (2.2)	5.3 (2.5)	7.7 (1.5)	4.0 (2.6)	7.8 (2.2)	<0.001^d^
Verbal fluency	6.8 (3.1)	10.4 (3.2)	12.7 (2.7)	9.6 (3.0)	12.3 (2.8)	<0.001^e^
Forward Digit Span	8.7 (3.0)	9.7 (2.6)	10.8 (2.2)	8.6 (2.4)	10.9 (2.1)	<0.001^f^
Backward Digit Span	3.8 (2.4)	5.8 (2.1)	6.2 (2.1)	3.9 (2.1)	6.2 (2.4)	<0.001^g^
Boston naming test	11.5 (3.0)	14.0 (1.1)	13.9 (1.0)	13.0 (1.7)	14.3 (1.1)	<0.001^h^

AD, Alzheimer’s disease; MCI, mild cognitive impairment; PDD, Parkinson’s disease dementia; PDND, non-demented Parkinson’s disease; HC, healthy controls; *APOE*, apolipoprotein E; MMSE, mini-mental state examination; UPDRS, Unified Parkinson’s Disease Rating Scale.

Data presented as mean and standard deviation in parentheses unless noted.

*Presented as number of patients and percentage in parentheses.

^a^PDND vs HC, p < 0.001; PDND vs MCI, p < 0.001; PDND vs PDD, p = 0.04; PDND vs AD, p < 0.001.

^b^HC vs PDD, p = 0.02; HC vs AD, p = 0.01.

^c^PDD vs HC, p = 0.01; PDD vs MCI, p = 0.047; PDD vs PDND, p = 0.048; PDD vs AD, p < 0.001; AD vs HC, p < 0.001; AD vs MCI, p < 0.001; AD vs PDND, p < 0.001

^d^MCI vs HC, p < 0.001; MCI vs PDD, p < 0.001; MCI vs AD, p < 0.001; PDND vs PDD, p < 0.001; PDND vs AD, p < 0.001.; PDD vs AD p < 0.001.

^e^HC vs MCI, p = 0.01; HC vs PDD, p = 0.003; HC vs AD, p < 0.001; MCI vs PDND, p = 0.016; MCI vs AD, p < 0.001; PDND vs PDD, p = 0.004; PDND vs AD, p < 0.001; PDD vs AD, p = 0.001.

^f^HC vs PDD, p = 0.004; HC vs AD, p < 0.001; PDND vs PDD, p = 0.029; PDND vs AD, p = 0.003.

^g^HC vs PDD, p = 0.001; HC vs AD, p < 0.001; MCI vs PDD, p = 0.006; MCI vs AD, p < 0.001; PDND vs PDD, p = 0.004, PDND vs AD, p < 0.001.

^h^HC vs AD, p < 0.001; MCI vs AD, p < 0.001; PDND vs AD, p < 0.001; PDD vs AD, p = 0.02.

### Neuropsychological evaluation

AD patients scored significantly worse than HC, MCI, and PDND subjects across all cognitive tests. Regarding PDD and AD, AD patients had significantly worse performances on MMSE, delayed recall, and category verbal fluency than did PDD patients (MMSE, p < 0.001; delayed recall, p < 0.001; verbal fluency, p < 0.001). MCI patients had worse performance on delayed recall and category verbal fluency, compared to HC (p < 0.001 and p = 0.01, respectively). PDD patients scored worse than PDND patients on MMSE, delayed recall, category verbal fluency, forward and backward digit span tests (MMSE, 0.048; delayed recall, < 0.001; category verbal fluency, 0.004; forward digit span, 0.029; backward digit span, 0.004).

### Plasma NFL levels

**Figure 1:**
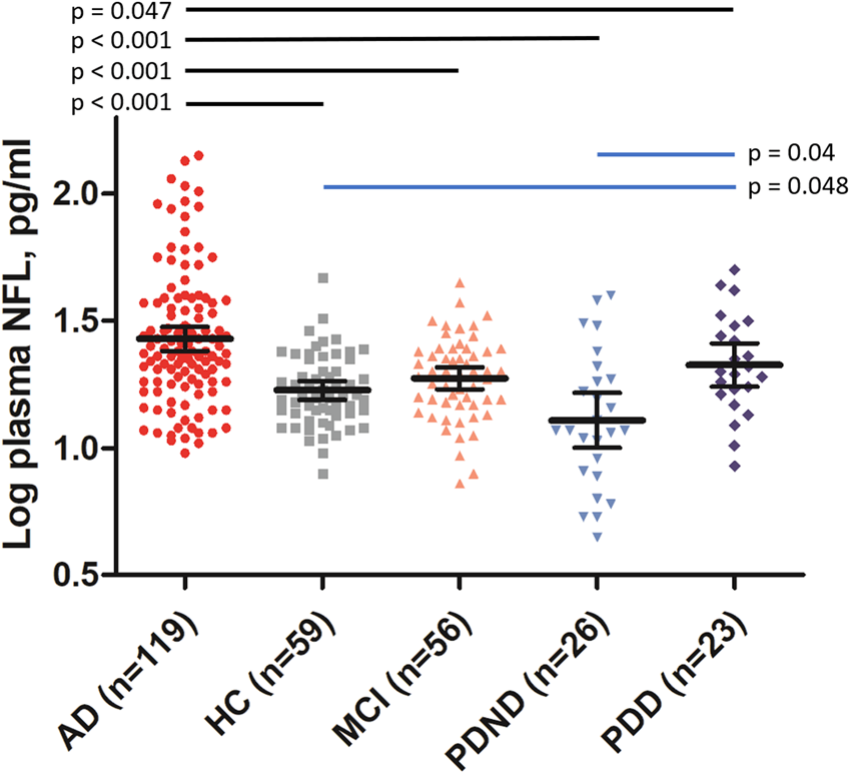
Comparison of plasma NFL levels among groups. Plasma NFL levels were higher in the AD group compared with controls, MCI group, PDND group, and PDD group. Plasma NFL levels were also higher in the PDD group, compared with controls and the PDND group. Abbreviations: NFL, neurofilament light chain; AD, Alzheimer’s disease; MCI, mild cognitive impairment; PDND, non-demented Parkinson’s disease; PDD, Parkinson’s disease dementia.

Mean plasma NFL level was 32.9 ± 25.5 pg/ml in AD patients, 20.0 ± 7.3 pg/ml in MCI patients, 15.4 ± 9.9 pg/ml in PDND patients, 23.3 ± 10.8 pg/ml in PDD patients, and 17.8 ± 6.4 pg/ml in controls. Male patients had higher plasma NFL levels than female patients (p = 0.03, t-test). Plasma NFL level increased with age (Pearson r = 0.427, p < 0.001). This age-related effect on plasma NFL level was seen across all sub-groups. After adjusting for age, sex, years of education, and *APOE* ɛ4 carrier status, AD patients had higher plasma NFL levels, compared with patients with HC, MCI, PDND (all p < 0.001), or PDD (p = 0.047) (Fig. 1 and Table 2). PDD patients had significantly higher plasma NFL levels, compared with PDND (p = 0.04) and control patients (p = 0.048). Plasma NFL levels were similar in MCI patients and PD patients, and also in MCI patients and PDND patients. When adjusting for age, sex, years of education, *APOE* ɛ4 carrier status and MMSE scores, AD patients still had higher plasma NFL levels compared with patients with HC (p = 0.0021) and PDND (p = 0.006) (Table 3).

**Table 2:** Results of one-way analysis of covariance in plasma NFL levels after adjusting age, sex, years of education, and *APOE* ɛ4 carrier status.

Parameter	B	SE	t value	P value
Intercept	0.454	0.171	2.661	0.008
Age	0.013	0.002	6.591	<0.001
Sex	−0.039	0.026	−1.504	0.134
Years of education	0.003	0.003	1.068	0.286
*APOE* ε4 carrier	0.027	0.029	0.918	0.359
Control	−0.201	0.034	−5.954	<0.001
MCI	−0.141	0.033	−4.254	<0.001
PDND	−0.225	0.048	−4.676	<0.001
PDD	−0.096	0.048	−1.996	0.047
AD	Ref			

Dependent variable: plasma NFL level (log transformation).

NFL, neurofilament light chain; *APOE*, apolipoprotein E; SE, standard error; MCI, mild cognitive impairment; PDND, non-demented Parkinson’s disease; PDD, Parkinson’s disease dementia; AD, Alzheimer’s disease.

**Table 3:** Results of one-way analysis of covariance in plasma NFL levels after adjusting age, sex, years of education, *APOE* ɛ4 carrier status, and MMSE scores.

Parameter	B	SE	t value	P value
Intercept	0.877	0183	4.798	<0.001
Age	0.011	0.002	5.473	<0.001
Sex	−0.042	0.025	−1.694	0.091
Years of education	0.006	0.003	2.249	0.025
*APOE* ε4 carrier	0.002	0.028	0.054	0957
MMSE	−0.014	0.003	−5.085	<0.001
Control	−0.091	0.039	−2.329	0.021
MCI	−0.049	0.037	−1.309	0.192
PDND	−0.137	0.049	−2.776	0.006
PDD	−0.035	0.048	−0.739	0.461
AD	Ref			

Dependent variable: plasma NFL level (log transformation).

NFL, neurofilament light chain; *APOE*, apolipoprotein E; SE, standard error; MMSE. Mini-mental status examination; MCI, mild cognitive impairment; PDND, non-demented Parkinson’s disease; PDD, Parkinson’s disease dementia; AD, Alzheimer’s disease.

### Plasma NFL level and MMSE score

Pooling all participants together, higher plasma NFL levels correlated with lower MMSE scores (Pearson r = −0.491, p < 0.001). In each diagnostic group, the correlation between plasma NFL level and MMSE score was strongly evident in AD patients (Pearson r = −0.370, p < 0.001), with a trend toward statistical significance in the MCI group (Pearson r = −0.216, p = 0.055). The correlation was also significant in all PD patients (Pearson r = −0.323, p = 0.023).

### Plasma NFL levels and UPDRS

Plasma NFL level was not correlated with UPDRS, among PDND patients (Pearson r = −0.069, p = 0.743), PDD patients (Pearson r = 0.056, p = 0.81), or among all PD patients (Pearson r = 0.177, p = 0.238).

### Plasma NFL level and *APOE* ε4 carrier status

Across all groups, plasma NFL levels were similar between *APOE* ε4 carriers and non-carriers (all participants, p = 0.22; AD group, p = 0.97, MCI group, p = 0.60; PDND group, p = 0.21; PDD group, p = 0.95; HC group, p = 0.95).

### Plasma NFL level and MCI conversion to AD

In MCI patients, we have followed 34 patients (34/45 = 60.7%), and the mean follow-up months is 19.5 ± 8 months. During follow-ups, 10 MCI patients have converted to AD. The plasma NFL levels were similar between the converter and non-converter in MCI patients (p = 0.26). The Cox regression model adjusting for age, sex, years of education, disease duration, and *APOE* ɛ4 carrier status also showed no association between NFL levels and MCI conversion to AD (Table 4).

**Table 4:** Cox regression analysis of predictor for MCI conversion to AD.

	HR	95% CI	P value
Age	1.04	0.87–1.24	0.69
Sex	1.29	0.28–5.89	0.74
Years of education	1.01	0.8–1.26	0.96
*APOE* ɛ4 carrier status	0.97	0.12–7.66	0.98
Disease duration	1.01	0.97–1.05	0.56
NFL	1.67	0.003–1108.73	0.88

HR, hazard ratio; *APOE*, apolipoprotein E; NFL, neurofilament light chain.

## Discussion

Our study found higher plasma NFL in PDD, compared with PDND patients. This finding indicates that plasma NFL level is associated with the cognitive function in PD patients. Among all parkinsonian disorders, typical PD is associated with the lowest elevation in plasma NFL levels, compared to HC^2,5^. Our results support this assertion. We also observed the greatest elevation in plasma NFL levels in patients with AD, compared to other patient groups. This finding is consistent with prior studies^1,22^ that showed elevated plasma NFL levels in AD, compared with MCI and HC. However, our results did not indicate that MCI patients had higher plasma NFL levels than HC. Analysis of the results collected for MCI and AD patients did not indicate that *APOE* ɛ4 carriers had higher plasma NFL than non-carriers. These findings are different from previous studies which suggested MCI patients had higher plasma NFL levels than HC and *APOE* ɛ4 carriers had higher plasma NFL than non-carriers^1^, which may reflect the highly nuanced and complex clinical entity in MCI^23^ and relatively low proportion of *APOE* ɛ4 carriers among the Asian AD and MCI groups^24–27^.

Compared with PDND patients, PDD patients had significantly higher levels of periventricular and deep white matter lesions (WMLs)^28^. Previous studies have reported an association between cerebral WMLs and cognitive dysfunction^29^. Among patients with WMLs, tasks that evaluate the speed of cognitive processes appear to be more affected by periventricular WMLs than are memory tasks^29^. According to the consensus in the contemporary scientific literature, executive dysfunction is greater in PDD patients than in PDND patients. Disruption in the dorsolateral prefrontal circuit is known to result in executive dysfunction^30^. The circuit consists of fibers that project from frontal cortex to deep structures in the basal ganglia. In PDD, in addition to degeneration of dopaminergic neurons in substantia nigra, periventricular WMLs may further interfere with executive function. Given that NFL may be a marker of white matter pathology^31^, PDD patients are expected to have higher plasma NFL levels than PDND patients. In addition to higher levels of periventricular and deep WML in PDD patients compared with PDND patients, the reduction of gray matter volume (GMV) over frontal-limbic-temporal regions might contribute to the higher NFL levels in PDD patents^32,33^. Although the paper did not show the association between the WMLs and plasma NFL level in AD patients^1^, some other papers reported that the NFL levels related to WML^34^. The reason increased NFL in cognitively impaired PD patients could be either increased WML or reduced GMV over frontal-limbic-temporal regions.

In the current study, we found that plasma NFL level was positively correlated with age, regardless of diagnostic status. This result corresponds to those reported previously^35–37^. Nonetheless, the reason for this association between age and plasma NFL level remains to be elucidated. Presumably, it could be due to age-related atrophic changes or brain hypometabolism, leading to NFL and axonal degeneration. In the present study, plasma NFL level correlated with male gender. However, the relationship between plasma NFL level and sex has not been consistently observed. Mattson *et al*.^1^ found no correlation, whereas Hansson *et al*.^2^ reported that women have higher NFL levels than men. Additional studies are needed to clarify the relationship between sex and NFL levels.

**Figure 2:**
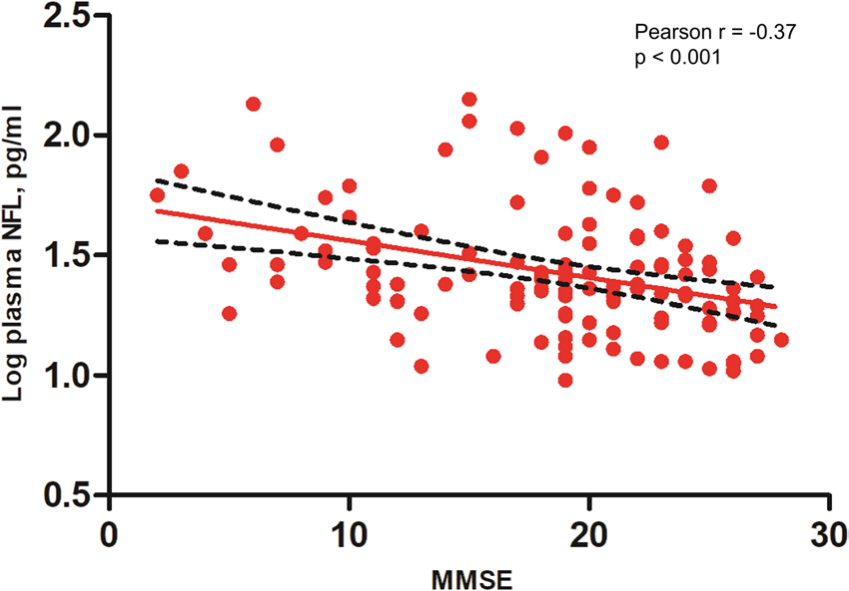
Correlation between plasma NFL and MMSE score in the AD group. Plasma NFL level correlates with MMSE score in the AD group (Pearson r = −0.37, p < 0.001). Abbreviations: NFL, neurofilament light chain; MMSE, mini-mental state examination; AD, Alzheimer’s disease; PDD, Parkinson’s disease dementia.

When the overall patient population was considered, plasma NFL level showed a negative association with MMSE score (Fig. 2). Such an association was previously reported^1^. In line with previous reports, the correlation was more significant in AD patients than in MCI and HC^22^. The reduced significance of the correlation between NFL levels and MMSE scores in the MCI group may reflect the limited range of MMSE scores allowed by the disease’s clinical definition. In addition, plasma NFL level may not reflect subtle cognitive changes that are detected by the MMSE during progression of MCI. In PD patients, plasma NFL levels were also associated with MMSE scores but no correlation between NFL level and UPDRS score was observed. The results suggested that plasma NFL might be a biomarker for cognition in AD and PD patients but not a biomarker for motor symptoms in PD patients. In addition, our data showed that plasma NFL levels were still higher in AD group compared with HC and PDND groups after adjusting MMSE scores, which suggested plasma NFL was a more specific biomarker for AD than PD.

The current study has some limitations. First, diagnoses of AD and MCI were made using only clinical criteria, without the use of biomarkers of Aβ deposition or tau-mediated neuronal degeneration. This factor may have impeded diagnostic accuracy. It was not possible for us to analyze relationships among biomarkers of Aβ deposition, tau-mediated neuronal degeneration, and plasma NFL levels. Second, the relatively small sample size of each diagnostic group is a concern. We performed a cross-disease comparison of MCI, AD, PDND, and PDD, which may serve as supplemental evidence for future clinical use of plasma NFL as a diagnostic biomarker.

## Conclusion

In conclusion, plasma NFL levels were significantly increased in the AD group, compared with controls and MCI, PDND, and PDD patients. Plasma NFL levels were significantly higher in the PDD group, compared with the PDND group. High plasma NFL correlated with poor cognition both in AD and PD patients, but not with motor symptoms in PD patients. Plasma NFL may represent a biomarker of cognitive decline in AD and PD, with greater specificity for AD. Future longitudinal follow-up studies are needed to validate whether plasma NFL levels may be used to predict progression of dementia in MCI and PD patients.

## Data Availability

The datasets used and/or analyzed during the current study are available from the corresponding author upon request.
